# Who let the dogs out?: detrimental role of Galectin-3 in hypoperfusion-induced retinal degeneration

**DOI:** 10.1186/s12974-015-0312-x

**Published:** 2015-05-14

**Authors:** Oscar Manouchehrian, Karin Arnér, Tomas Deierborg, Linnéa Taylor

**Affiliations:** Department of Ophthalmology, BMC, Lund University, Klinikgatan 26, Lund, S-22184 Sweden; Experimental Neuroinflammation Laboratory, BMC, Lund University, Klinikgatan 26, Lund, S-22184 Sweden

**Keywords:** Retina, Microglia, Galectin 3, Müller cells, Neuroinflammation, Gliosis, Ischemia

## Abstract

**Background:**

Retinal ischemia results in a progressive degeneration of neurons and a pathological activation of glial cells, resulting in vision loss. In the brain, progressive damage after ischemic insult has been correlated to neuroinflammatory processes involving microglia. Galectin-3 has been shown to mediate microglial responses to ischemic injury in the brain. Therefore, we wanted to explore the contribution of Galectin-3 (Gal-3) to hypoperfusion-induced retinal degeneration in mice.

**Methods:**

Gal-3 knockout (Gal-3 KO) and wildtype (WT) C57BL/6 mice were subjected to chronic cerebral hypoperfusion by bilateral narrowing of the common carotid arteries using metal coils resulting in a 30% reduction of blood flow. Sham operated mice served as controls. After 17 weeks, the mice were sacrificed and the eyes were analyzed for retinal architecture, neuronal cell survival, and glial reactivity using morphological staining and immunohistochemistry.

**Results:**

Hypoperfusion caused a strong increase in Gal-3 expression and microglial activation in WT mice, coupled with severe degenerative damage to all retinal neuronal subtypes, remodeling of the retinal lamination and Müller cell gliosis. In contrast, hypoperfused Gal-3 KO mice displayed a retained laminar architecture, a significant preservation of photoreceptors and ganglion cell neurons, and an attenuation of microglial and Müller cell activation.

**Conclusion:**

Moderate cerebral blood flow reduction in the mouse results in severe retinal degenerative damage. In mice lacking Gal-3 expression, pathological changes are significantly attenuated. Gal-3 is thereby a potential target for treatment and prevention of hypoperfusion-induced retinal degeneration and a strong candidate for further research as a factor behind retinal degenerative disease.

**Electronic supplementary material:**

The online version of this article (doi:10.1186/s12974-015-0312-x) contains supplementary material, which is available to authorized users.

## Introduction

Ischemia, a pathological condition caused by inadequate blood flow, is a highly deleterious condition when affecting the central nervous system. In patients, chronic cerebral hypoperfusion is a common cause of cognitive impairment and a key characteristic of pathologies such as vascular dementia [1,2]. This type of insult is characterized by progressive neuronal degeneration, gliosis, and diffuse demyelination; however, the mechanism behind this progression remains unclear.

In addition to neurological impairment in the brain, chronic cerebral hypoperfusion induced by stenosis or occlusion of the common carotid arteries has been found to lead to retinal and optic nerve degeneration in several experimental animal models [2-6]. Similar to the progressive pathological process seen in the brain, retinal degeneration following chronic occlusion of the common carotid arteries occurs in several stages. Microglial activation has been observed within the first 24 h, apoptosis of retinal ganglion cells after 1 week, followed by death of inner retinal neurons after 2 months and photoreceptors after 4 months [3]. Similar results have been obtained after transient occlusion of the middle cerebral artery, which has been found to cause a decrease in retinal function, as well as gliosis and ganglion cell death [7]. The microglial activation is a prominent feature of infarct lesions resulting from ischemic insults in the brain [8]. Uncontrolled activation and inflammation is thought to be one of the major contributing factors to the progressive neurodegeneration and resulting neurological impairment seen in ischemia [9]. Although microglial reactivity has been shown to have some beneficial effects, prolonged and dysregulated activation can exacerbate the pro-inflammatory process and thereby increase neurotoxicity [10]. Retinal neuroinflammation is a relatively new area of research, and the correlation between inflammatory factors and detrimental processes such as gliosis during injury and disease has so far been largely unexplored.

One of the mediators of microglial activation and inflammation during ischemic brain injury is the carbohydrate-binding protein Galectin-3 (Gal-3) [11]. Previous studies have shown that Gal-3 is required for microglial activation, and that it can contribute to the early inflammatory process, as well as enhancing post-ischemic tissue remodeling [11,12]. Recently, we demonstrated that Galectin-3 is a key player in alpha-synuclein-induced inflammation, relevant to Parkinson’s disease [13]. In the retina, increased Gal-3 expression has been implicated in the pathological process in diabetic retinopathy, light-induced degeneration, and retinal detachment [14,15]. However, its contribution to ischemic injury has so far not been elucidated.

For this study, our hypothesis was that Gal-3 is a mediator of neuroinflammation and neuronal cell death in hypoperfusion-induced retinal degeneration. Using a previously established microcoil carotid stenosis model in Gal-3 null mice with a pure C57BL/6 background as well as C57BL/6 wildtype mice, we explore ischemic damage in the retina.

## Material and methods

### Animals

Gal-3 null mice [16] with a pure C57BL/6 background were obtained originally from Dr. K. Sävman from the Gothenburg University and bred at the Lund University (Sweden). Mice were housed under a 12 h light/12 h dark cycle with access to food and water *ad libitum*. All procedures were carried in accordance with the international guidelines and were approved by the Malmö-Lund Ethical Committee for Animal Research in Sweden (M425-12).

The operation procedure has been well described previously [2] and will be summarized here. Age-matched male mice (5 to 8 months wildtype mice, C57BL/6 (wildtype (WT), *n* = 17) and Gal-3 knockout mice (*n* = 18) with C57BL/6-background were used. Both WT and KO mice were generated from littermate breeding couples to minimize genetic variation between the WT and KO mice. WT mice (*n* = 12) and Gal3-KO mice (*n* = 12) were subjected to hypoperfusion of the brain (WT hypo). Sham operations were also performed, WT (*n* = 5) and Gal3-KO (*n* = 6). For the hypoperfusion and sham operations, mice were anesthetized with 5% isofluorane and anesthesia was maintained at 2% isofluorane in oxygen. The common carotid arteries were exposed with a small neck incision. For hypoperfusion, metal coils (wire diameter of 0.08 mm; inner diameter (ID): 0.18 mm; pitch : 0.50 mm; total length: 2.5 mm; surface: Au-coated (Invitrotech Co., LTD, Shimogasa-cho Kusatsu, Shiga, Japan) were encircled onto the common carotid arteries, reducing blood flow to about 70% [2]. Anesthesia was discontinued after 15 min, and the wound was sealed and locally anesthetized with Marcain (Bupivacaine, Apoteket, Umeå, Sweden) 1.25 mg/kg. The sham operated mice were exposed to the same procedure but had no coils inserted. 17 weeks post surgery, the animals were sacrificed using 5% isofluorane and the eyes enucleated. Immediately after enucleation, the eyes were fixed in 4% paraformaldehyde in 0.1 M phosphate buffer, pH 7.2 for 4 h at 4°C.

### Histology

Histological examinations were performed as previously described [17], and only briefly recapped here. After fixation, the eyes were macroscopically inspected, and infiltrated with 0.1 M Sörensens medium with increasing concentrations of sucrose, up to 25%, for cryoprotection. They were then embedded in egg albumin/gelatine medium for cryosectioning at −20°C with a section thickness of 12 μm. For light microscopy, every 10th slide was stained with hematoxylin and eosin (HTX). For immunohistochemical labeling, adjoining slides were chosen. The specimens were rinsed 3 times with PBS containing 0.1% Triton- X, and then incubated with PBS containing 1% bovine serum albumin (BSA) for 20 minutes at room temperature. After this, the specimens were incubated overnight at 4°C with the respective primary antibody (Table 1). In the double labeling for glutamine synthetase (GS)/bFGF, NeuN/Recoverin, Gal-3/Iba-1, Gal-3/glial fibrillary acidic protein (GFAP) and Gal-3/cellular retinaldehyde-binding protein (CRALBP) both primary antibodies were added at this stage. The specimens were then rinsed in PBS-Triton-X (0.1%) and incubated for 45 min with a secondary fluorescein isothiocyanate (FITC) or Texas Red-conjugated antibody (Table 1). In the double labeling for GS/bFGF, NeuN/Recoverin, Gal-3/Iba-1, Gal-3/GFAP, and Gal-3/CRALBP both secondary antibodies were added at this stage. The specimens were then mounted in Vectashield mounting medium with 4′,6-diamidino-2-phenylindole (DAPI; Vector laboratories Inc., CA, USA). Negative control experiments were performed as above, replacing the primary antibody with PBS containing 1% BSA. Normal adult mouse retina was used as a positive control.

**Table 1:** Table of primary and secondary antibodies used for immunohistochemical analysis

	**Antibody name**	**Target cell**	**Species**	**Dilution**	**Source**
Antigen					
Neuronal nuclei (NeuN)	Anti-neuronal nuclei	Ganglion cells	Mouse monoclonal	1:100	Millipore, USA
Glutamine synthetase (GS)	Rabbit anti-GS	Müller cells	Rabbit polyclonal	1:200	Abcam, Cambridge, UK
Recoverin	Anti-recoverin	Rod and cone photoreceptors	Rabbit polyclonal	1:10,000	Chemicon International, CA, USA
Rhodopsin	Rho4D2	Rod photoreceptor	Mouse monoclonal	1:100	Kind gift of Prof. RS Molday, Vancouver, Canada
Iba-1	Rabbit anti-Iba-1	Microglia	Rabbit polyclonal	1:500	Wako Pure Chemical Industries Ltd., Osaka, Japan/Nordic biolabs
Galectin-3	Galectin-3 antibody	Mainly microglia	Rat	1:300	Hakon Leffler
Calbindin	Anti-calbindin D-28K	Horizontal cells	Mouse monoclonal	1:200	Sigma Aldrich, St Louis, MO, USA
Glial fibrillary acidic protein (GFAP)	Anti-glial fibrillary acidic protein	Activated Müller cells, astrocytes	Mouse monoclonal	1:200	Chemicon International, CA, USA
PKCpan	Phospho-PKC (pan)	Rod bipolar cells	Rabbit polyclonal	1:200	Cell Signaling, Beverly, MA, USA
Cellular retinaldehyde-binding protein (CRALBP)	Anti-CRALBP [B2]	Müller cells	Mouse monoclonal	1:200	Chemicon International, CA, USA
Secondary antibody					
Fluorescein isothiocyanate (FITC)	Anti-mouse IgG FITC conjugate	Anti-mouse	Goat	1:200	Sigma Aldrich, St Louis, MO, USA
Texas red	Texas red dye-conjugated AffiniPure	Anti-rabbit	Donkey	1:200	Jackson ImmunoResearch, PA, USA

### Microscopy and image analysis

The histological sections and immunohistochemically labeled specimens were examined using an epifluorescence microscope (Axiophot; Zeiss, Oberkochen, West Germany) equipped with an Olympus digital camera system (Olympus, Tokyo, Japan) and a digital acquisition system (DP 70; Olympus, Tokyo, Japan). Double-labeled specimens were viewed using an optical and epifluorescence microscope (Axio Imager M2, Carl Zeiss Microscopy GmbH, Jena, Germany) equipped with a digital camera system (AxioCam MRm, Carl Zeiss) and a digital acquisition system (ZEN 2012 blue edition, Carl Zeiss). Confocal images of Iba1/Gal-3 and Gal-3/CRALBP labelings were obtained using a Nikon A1 Confocal on a Ti-E microscope (Nikon, Chiyoda-ku, Tokyo, Japan) and processed using NIS-Elements (Nikon, Chiyoda-ku, Tokyo, Japan). Photographs for panels were taken centrally. Images were viewed and processed using Photoshop (Adobe Systems, Mountain View, CA, USA).

### Statistical analysis

To statistically quantify survival of individual cell types, sections derived from HTX as well as immunohistochemistry were used. Ganglion cell and photoreceptor survival, as well as bipolar cell length, was analyzed using cell counts in NeuN labelings, labeled cell rows in recoverin labelings, and length measurements in PKCpan labelings. These analyses were performed as previously described [18-20]. In brief, one central photograph and one at the mid-periphery (two central and two from mid-periphery with PKCpan), taken using a fixed exposure time, were analyzed per retina from a central section (including the optic nerve). Cells in the ganglion cell layer (GCL) labeled with NeuN (ganglion cells) and vertical cells rows labeled with recoverin (photoreceptors) were counted, and the length of PKCpan-labeled bipolar cells was measured at 40× magnification. For photoreceptor rows, two rows were counted per image. For PKCpan width, one bipolar cell length was counted per image. All retinal sections were processed in the same batch for each immunohistochemical staining. Fluorescence intensity was used as a tool to quantify GFAP, GS, Iba1, and Galectin-3 labeling and was analyzed using ImageJ (Rasband, W.S., ImageJ, U. S. National Institutes of Health, Bethesda, Maryland, USA, http://imagej.nih.gov/ij/, 1997 to 2014). For each image, a rectangular area (fixed for each batch) was analyzed, placed within the area of interest, that is, from the NFL to outer limiting membrane, ensuring the measurements were not influenced by autofluorescence from inner and outer segments. The background, obtained from negative controls, was then subtracted, and the mean fluorescence measurement was noted as previously described [21]. Data were analyzed using two-way ANOVA with a Tukey post hoc test (GraphPad InStat; GraphPad Software, San Diego, CA, USA). Raw data from intensity measurements and cell counts were used to generate mean values for each of the different groups. Values of *P* < 0.05 were considered significant.

## Results

### Morphology

**Figure 1:**
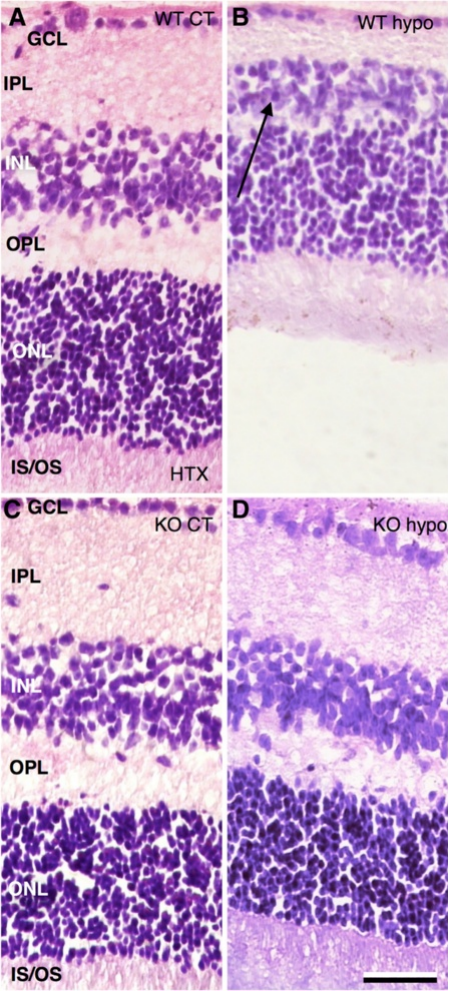
Hematoxylin and eosin staining of retinal sections. **(A)** WT control animals reveal clearly defined nuclear and plexiform layers. **(B)** WT hypo mouse retinas reveal thin and highly disorganized cell and plexiform layers with numerous pyknotic cells (arrow).The ganglion cell layer appear thin and is populated by small cell bodies. The smaller picture shows a central section of a WT hypo specimen with almost no survival. **(C)** KO control mice display clearly defined nuclear and plexiform layers. **(D)** Hypoperfused Gal-3 knockout mouse retinas (KO hypo) show a clearly laminated architecture with well populated cell layers. Scale bar = 50 μm.

Macroscopic inspection revealed no retinal neovascularization or retinal hemorrhages. Hematoxylin and eosin staining of WT and Gal3-KO controls, as well as Gal3-KO hypoperfused retinas, revealed clearly defined nuclear and plexiform layers (Figure 1A,C,D). In contrast, hypoperfused wildtype mouse retinas (WT hypo) revealed thin and highly disorganized nuclear layers with numerous pyknotic cells, as well as a thinning of the inner plexiform layer (IPL) and an almost complete loss of the outer plexiform layer (OPL; Figure 1B). The ganglion cell layer was populated by small cell bodies.

### Immunohistochemistry

**Figure 2:**
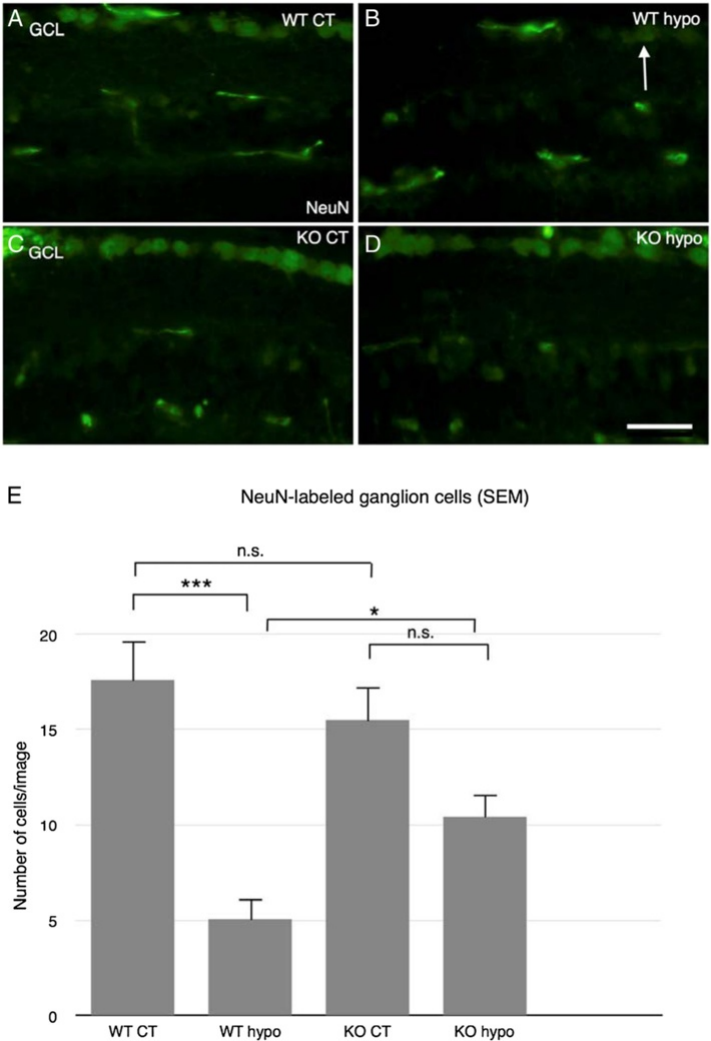
Immunohistochemical labeling of ganglion cells. **(A)** WT control retinas reveal large cell bodies of ganglion morphology in the GCL. **(B)** WT hypo mice show only isolated cell bodies in the GCL. **(C)** KO control animals show plentiful large cell bodies of ganglion morphology in the GCL. **(D)** KO hypo retinas reveal large cell bodies in the GCL. Scale bar = 50 μm. **(E)** Statistical analysis of NeuN-labeled ganglion cells in the GCL. No significant difference between WT and KO control animals. Significantly fewer ganglion cells in WT hypo animals compared to corresponding controls and KO hypo retinas (*P* < 0.001; *P* < 0.05, respectively). Error bars SEM. *P < 0.05; **P < 0.01; ***P < 0.001.

NeuN labeling revealed numerous large cell bodies of ganglion morphology in the GCL in both WT and KO controls, with no significant difference in the number of cells (Figure 2A,C,E). In WT hypo specimens, only isolated cell bodies were seen (Figure 2B). In contrast, KO hypo counterparts revealed significantly more labeled cell bodies present in the GCL (Figure 2D,E; *P* < 0.05). WT hypo specimens also had significantly fewer ganglion cells compared to corresponding controls (*P* < 0.001; Figure 2E).

**Figure 3:**
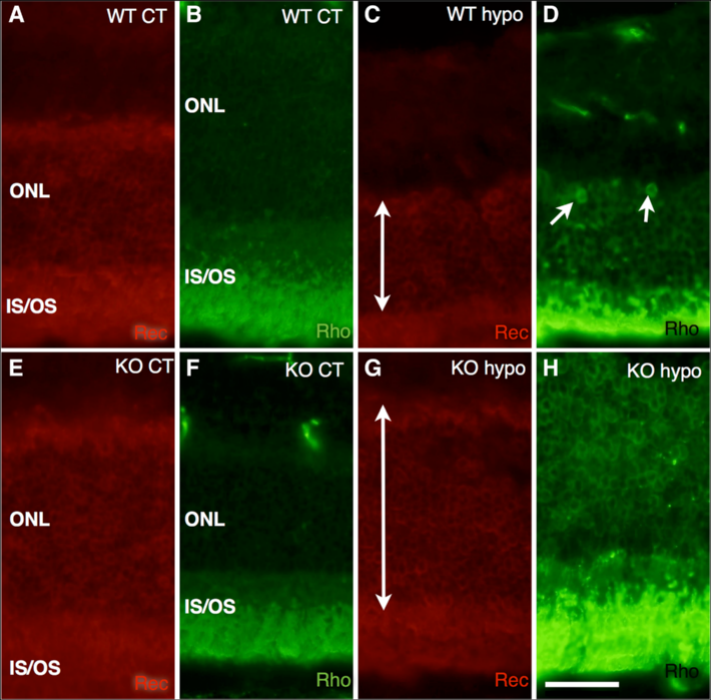
Immunohistochemical labeling of photoreceptor cells. **(A)** Recoverin-labeled WT control retinas reveal labeling of outer segment at the outer border of the retina, as well as weaker labeling of inner segments and photoreceptor cell bodies. **(B)** Rhodopsin labeling of WT control animals shows inner and outer segments as well as photoreceptor cell bodies in the ONL. **(C)** WT hypo retinas display recoverin labeling of disorganized inner and outer segments as well as labeling of photoreceptor cell bodies. **(D)** WT hypo specimens display strong rhodopsin labeling of outer segments and labeling of inner segments and photoreceptor cell bodies. The specimens show a disorganized appearance as well as displaced rhodopsin expression to cell bodies in the outer nuclear layer (arrows). **(E)** KO control retinas reveal recoverin labeling of inner and outer segments as well as photoreceptor cell bodies. **(F)** Rhodopsin labeling of KO control animals shows strong labeling of inner and outer segments and weaker labeling of photoreceptor cell bodies. **(G)** Recoverin-labeled KO hypoperfused retinas display labeling of inner and outer segments as well as photoreceptor cell bodies. **(H)** KO hypo mice reveal very strong labeling of the outer segments and weaker labeling of inner segments. Some displacement of rhodopsin expression to cell bodies in the ONL is observed. Scale bar = 50 μm.

**Figure 4:**
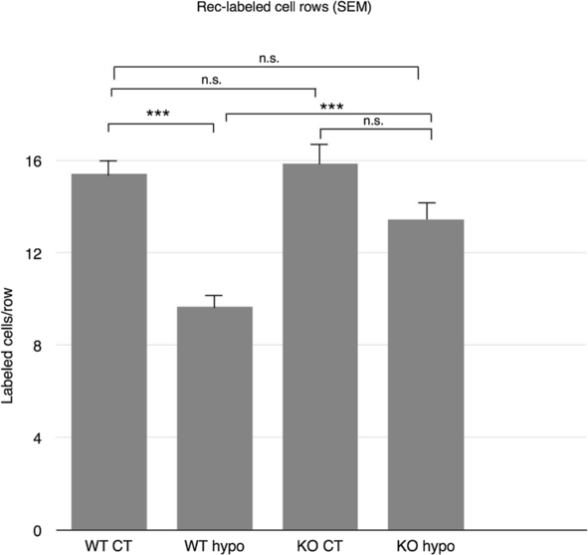
Statistical analysis of recoverin-labeled vertical photoreceptor cell rows. No significant difference is seen between WT control, KO control, and KO hypo retinas. Significantly fewer ONL cell rows are found in WT hypo specimens compared to KO hypo retinas as well as corresponding controls (*P* < 0.001). Error bars SEM. *P < 0.05; **P < 0.01; ***P < 0.001.

In both WT and KO control retinas, recoverin and rhodopsin labelings were present in photoreceptor outer segments (OS) at the outer border of the retina, as well as weaker labeling of inner segments (IS) and photoreceptor cell bodies (Figure 3A,B,F,G). WT hypo specimens displayed recoverin labeling similar to that seen in corresponding controls, albeit with significantly fewer labeled cell rows present (*P* < 0.001; Figures 3C and 4). In these specimens, higher rhodopsin labeling intensity was found in the IS/OS as well as displaced rhodopsin expression in scattered photoreceptor cell bodies (Figure 3D). Similarly, recoverin-labeled KO hypo retinas were comparable to the corresponding controls with no significant difference in the number of labeled cell rows, although an increase in rhodopsin labeling of IS/OS and cell bodies was observed (Figures 3G,H and 4).

**Figure 5:**
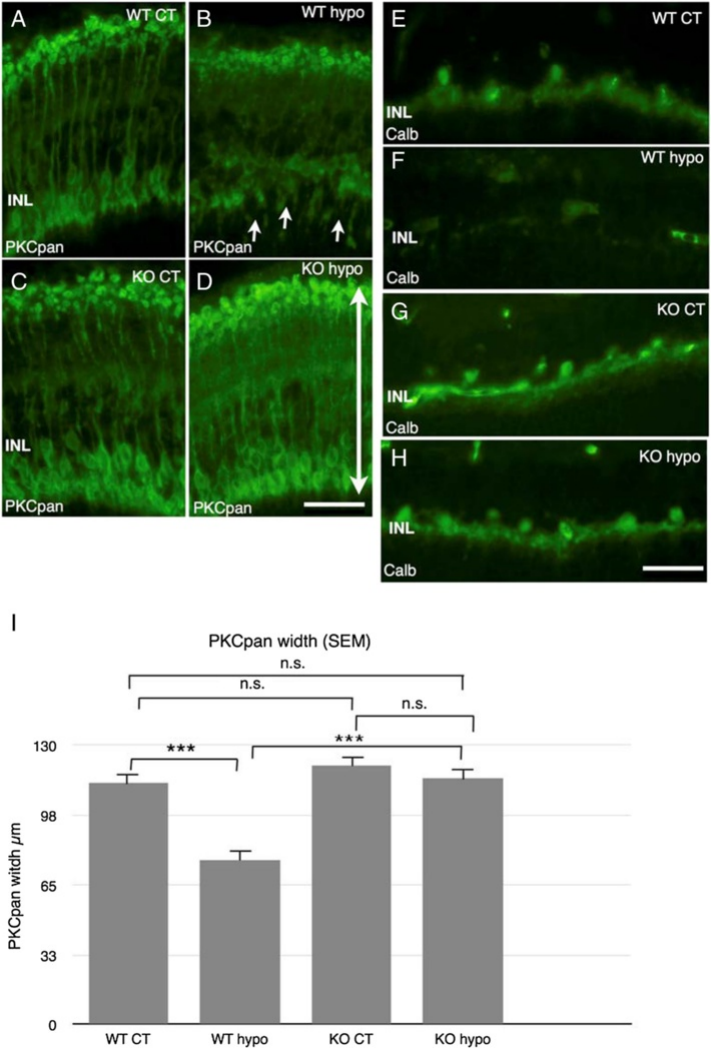
Immunohistochemical labeling of bipolar cells (PKCpan), horizontal cells (Calbindin). A-D PKCpan. E-H Calbindin. **(A)** WT control animals show numerous well organized bipolar cells and their processes in the INL and IPL, with strong labeling intensity present in the cell bodies in the INL. **(B)** WT hypo retinas display short bipolar cells with disorganized cell bodies and terminals and sprouting processes in the ONL (arrows). **(C)** KO control retinas show a multitude of well organized bipolar cells and their processes in the INL and IPL, respectively. Strong labeling is present in the terminals in the IPL and the cell bodies in the INL. **(D)** KO hypo specimens display numerous bipolar cells and processes in the INL and IPL, with retained organization and no sprouting. **(E)** WT control retinas reveal horizontal cell bodies and their processes in the INL and OPL, respectively. **(F)** WT hypo animals, no labeled cells are seen; however, scattered labeling of unidentified structures is present. **(G)** KO control animals display horizontal cell bodies and their processes in the INL and OPL. **(H)** KO hypo specimens reveal horizontal cell bodies and their processes in the INL and OPL. Scale bars = 50 μm. **(I)** Statistical analysis of PKCpan width (bipolar cell length) in the INL and OPL. Significantly shorter bipolar cells in WT hypo animals compared to corresponding controls and KO hypo retinas (*P* < 0.001 for both comparisons). Error bars SEM. *P < 0.05; **P < 0.01; ***P < 0.001.

PKCpan labeling of WT and KO controls showed strong labeling of a multitude of well organized bipolar cells and their processes in the inner nuclear layer (INL) and IPL, respectively (Figure 5A,C). In contrast to the control specimens, WT hypo specimens displayed shortened bipolar cells with disorganized cell bodies and terminals and sprouting processes, a behavior indicative of photoreceptor degeneration, in the outer nuclear layer (ONL) (*P* > 0.001; Figure 5B,I) [22]. In KO hypo counterparts, bipolar cells retained their organization and displayed no sprouting, as well as displaying significantly preserved cell span compared to WT hypo specimens (*P* > 0.001; Figure 5D,I). Calbindin labeling of WT and KO control retinas revealed horizontal cell bodies and their processes in the INL and OPL, respectively (Figure 5E,G). In WT hypo counterparts, only weak scattered labeling of unidentified structures was found (Figure 5F). In contrast, KO hypo retinas revealed labeling similar to that seen in control specimens (Figure 5H).

**Figure 6:**
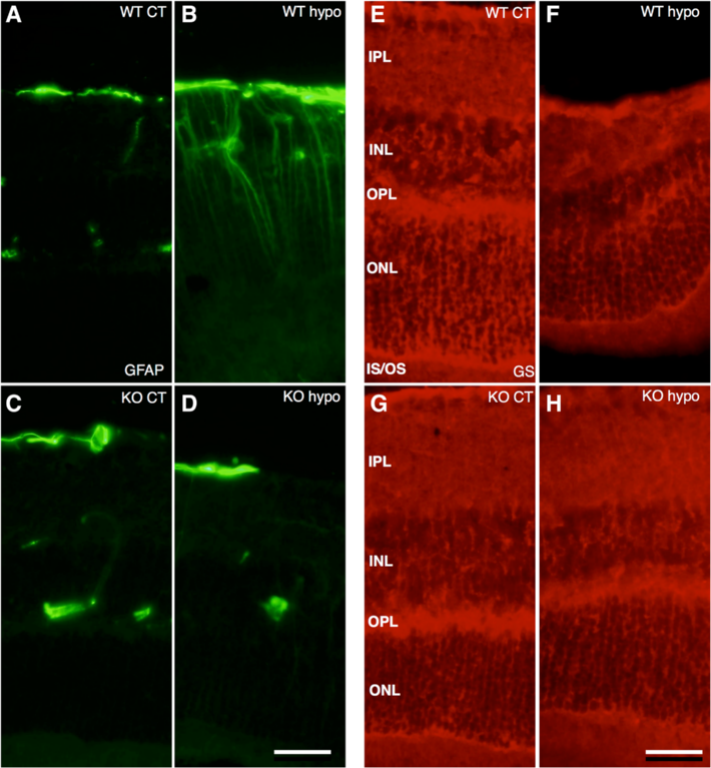
Immunohistochemical labeling of activated Müller cells and astrocytes (GFAP) and Müller cell (GS). **(A**-**D)** GFAP. **(E**-**H)** GS. (A) WT control retinas show scattered astrocytes and Müller cell endfeet present at outer border of the specimens, as well as isolated Müller cell processes in the IPL. Isolated retinal vessels are labeled in the INL. (B) WT hypo specimens display strong labeling of hypertrophic Müller cell fibers spanning the entire vertical width of the retina. (C) KO control specimens display labeling of scattered astrocytes and Müller endfeet at the outer border of the specimens and isolated blood vessels in the inner layers. (D) KO hypo mice show a discontinuous band of labeled astrocytes and Müller endfeet at the inner border, as scattered retinal vessels in the inner layers. (E) WT control animals reveal strong GS labeling throughout the specimens. (F) In WT hypo mice, GS labeling is present throughout the whole retina. (G) KO control animals reveal strong GS labeling throughout the retina. (H) Hypoperfused KO mice display GS labeling throughout the retina. Scale bars = 50 μm.

**Figure 7:**
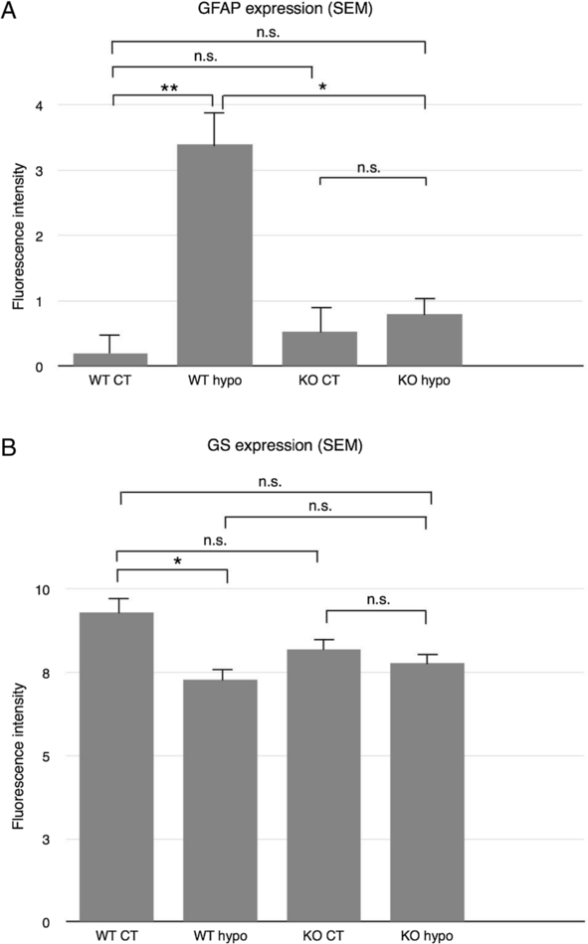
Statistical analysis of GFAP and GS labeling fluorescence intensity. **(A)** Fluorescence intensity of GFAP labeling measured by ImageJ. No significant difference is observed between WT controls, KO control, and KO hypo specimens. Hypoperfused wildtype animals show a significantly higher fluorescence intensity compared to KO hypo specimens as well as the corresponding controls (*P* < 0.01; *P* < 0.05), respectively. Error bars SEM. **(B)** Fluorescence intensity of GS labeling measured by ImageJ. No significant difference in fluorescence intensity is found between WT control, KO control, and KO hypo specimens. Significantly lower fluorescence intensity is seen in hypoperfused wildtype retinas compared to the corresponding controls (*P* < 0.05). Error bars SEM. *P < 0.05; **P < 0.01; ***P < 0.001.

In both WT and KO control retinas, GFAP labeling revealed scattered astrocytes and Müller cell endfeet present at outer border of the specimens, as well as isolated Müller cell processes in the INL and ONL, with no significant difference in labeling intensity (Figures 6A,C and 7A). Strong labeling of vertical, hypertrophic Müller cell fibers spanning the entire width of the retina was present in WT hypo specimens (Figure 6B). In contrast, labeling of KO hypo retinas appeared similar to the corresponding controls (Figure 6D). GFAP fluorescence intensity was significantly higher in WT hypo animals compared to their corresponding controls and KO hypo specimens (*P* < 0.01 and *P* < 0.05, respectively, Figure 7A). GS labeling was found throughout the specimen in both WT and KO control retinas, with no significant difference in fluorescence intensity (Figures 6E,G and 7B). Hypoperfused animals in both groups displayed a similar labeling pattern (Figure 6F,H), although the fluorescence intensity was found to be significantly lower in the WT retinas compared to the corresponding controls (*P* < 0.05; Figure 7B).

**Figure 8:**
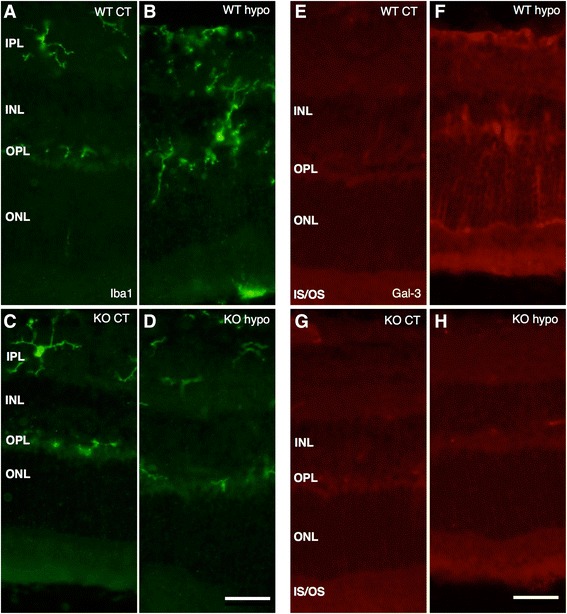
Immunohistochemical labeling of microglia and Gal3 expression. **(A**-**D)** Iba1. **(E**-**H)** Gal3. (A) Iba1-labeled WT control mice show scattered, ramified microglia present in the plexiform layers. (B) Hypoperfused WT animals display numerous microglia of both ramified and amobeoid morphology present throughout the retina, with isolated amobeoid cells in the outermost layers. (C) KO control retinas display ramified microglial cells in the plexiform layers. (D) A similar labeling pattern and cell morphology is seen in KO hypo mice. (E) WT control animals show no Gal-3 labeling. (F) Hypoperfused WT retinas show striated labeling spanning all nuclear layers with strongly labeled scattered structures in the inner layers. (G) KO control animals reveal no labeling of Gal-3. (H) Hypoperfused KO mice display no labeling of Gal-3. Scale bars = 50 μm.

**Figure 9:**
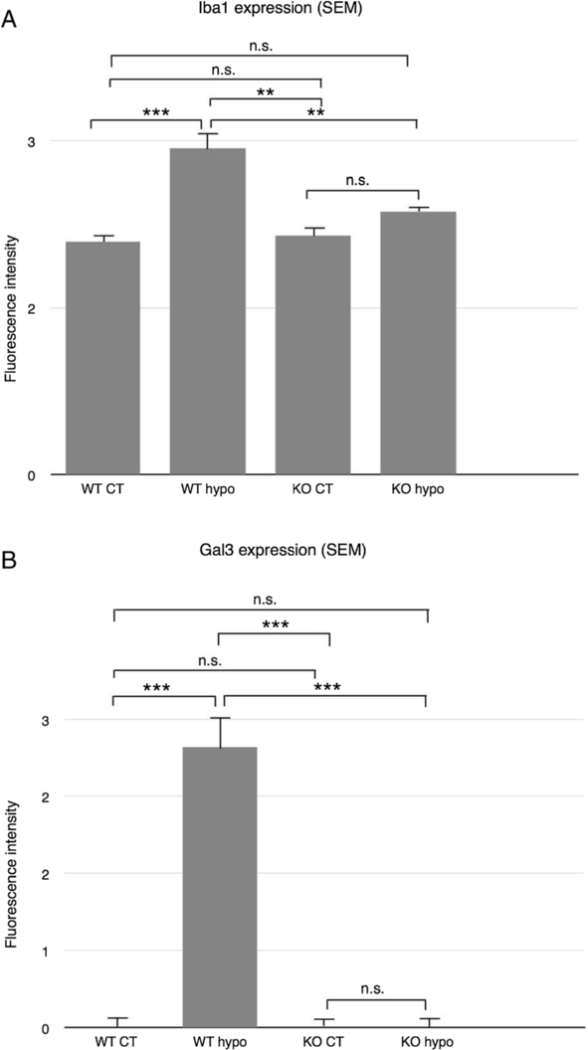
Statistical analysis of Iba-1 and Gal3 labeling fluorescence intensity. **(A)** Fluorescence intensity of Iba-1 labeling measured by ImageJ. No significant difference between WT control, KO control, and KO hypo retinas. WT hypo animals show significantly higher fluorescence intensity compared to all other groups (*P* < 0.001; *P* < 0.01; *P* < 0.01, respectively). Error bars SEM. **(B)** Fluorescence intensity of Gal-3 labeling measured by ImageJ. No significant difference between WT control, KO control, and KO hypo specimens. Hypoperfused wildtype animals reveal a significantly higher fluorescence intensity compared to all other groups (*P* < 0.001 for all comparisons). Error bars SEM. *P < 0.05; **P < 0.01; ***P < 0.001.

**Figure 10:**
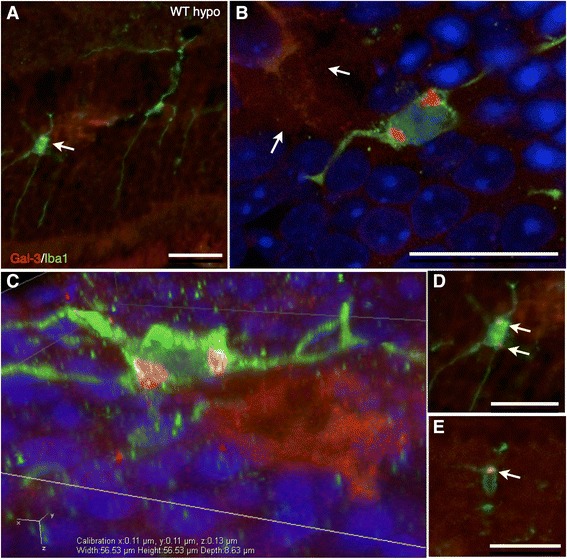
Immunohistochemical labeling of Gal-3 (red), Iba-1 (green), and DAPI (blue), epifluorescent imaging and confocal imaging. **(A)** Double-labeled WT hypo retinas with Gal-3/Iba-1 show colocalized expression of Gal3 in small, round structures in Iba1-labeled microglial cell bodies (arrow). **(B)** Confocal linescan z-stack slice revealing two extra-nuclear Gal-3 inclusions in a microglial cell, adjacent to a large, non-uniform Gal-3 labeled structure (arrows). **(C)** A three-dimensional rendered image of a microglial cell with two Gal-3 inclusions, adjacent to a Gal-3 positive structure. **(D)** Colocalized labeling of Gal-3 inclusions (arrows) in an Iba1-positive microglial cell. **(E)** Iba1-positive cell with Gal3-labeled structure (arrow). Scale bars = 25 μm.

**Figure 11:**
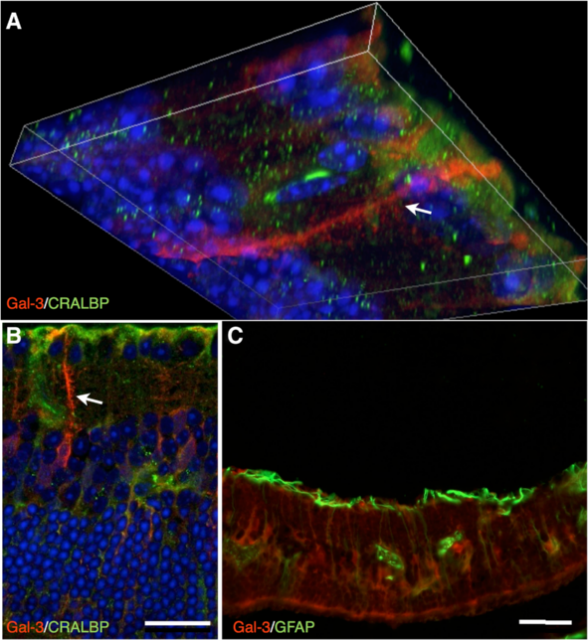
Immunohistochemical labeling of Gal-3, with Müller cell markers GFAP and CRALBP, and DAPI; epifluorescent imaging and confocal imaging of WT hypoperfused retinas. **(A)** A confocal linescan z-stack image of a double labeling of Gal-3/CRALBP show Gal-3 labeling of large structures vertically spanning the inner layers (arrow), as well as scattered structures. No colocalized labeling of CRALBP and Gal-3 was found. **(B)** Confocal image of Gal-3/CRALBP double labeling displays no colocalization. Large, vertical Gal-3 positive structures are present (arrow). **C**) Gal-3/GFAP double labeling reveals no colocalization. Scale bars = 50 μm.

Iba1 labeling of WT and KO control retinas was used to reveal parenchymal cells as well as possible blood-born macrophages and showed cells of a ramified microglial morphology present mainly in the plexiform layers (Figure 8A,C), with no significant difference in labeling intensity between the two groups (Figure 9A). Hypoperfused WT specimens revealed numerous ramified and amoeboid cells resembling microglia present throughout the retina, with isolated amoeboid cells present in the outermost layers (Figure 8B). The fluorescence intensity was significantly higher compared to that of corresponding control and KO hypo retinas (*P* < 0.001; Figure 9A). KO hypo specimens appeared similar to control retinas with no significant difference in labeling intensity compared to the controls (Figures 8E and 9A). No Gal3 labeling was found in WT and KO control animals, with no difference in labeling intensity (Figures 8E,G and 9B). In contrast, the WT hypo retinas displayed strong, irregular Gal-3 labeling of structures scattered throughout all three nuclear layers (Figure 8F), with a significantly higher labeling intensity compared to WT control specimens (*P* < 0.001; Figure 9B). Labeling of KO hypo animals appeared similar to, and did not significantly differ in intensity from, that found in the corresponding controls (Figures 8H and 9B). A double labeling of Gal-3 and Iba1 revealed colocalization of small, round Gal-3-positive structures in the cell bodies of microglia in the WT hypo specimens (Figure 10A,B,C,D,E and Additional file 1). In addition, Iba1-labeled microglia were most often seen adjacent to Gal-3 positive structures resembling debris (Figure 10A,B,C). No colocalization of Iba1 and Gal-3 was found in any of the other groups (not shown). Double labelings of Gal-3/CRALBP and Gal-3/GFAP revealed no colocalization of these two proteins in WT hypo retinas (Figure 11A,B,C), not in any of the other groups (not shown).

## Discussion

### Summary

In this study, we have explored the contribution of Galectin-3 to the retinal degeneration observed in a chronic carotid stenosis model, which in the brain has been used as a model of vascular dementia [2-6]. Previous studies have described progressive retinal degeneration as a result of hypoperfusion through the common carotid arteries in several animal models, even after baseline blood flow has been restored [4,23-25]. This pathological process is associated with an increased GFAP expression in Müller cells, one of the hallmarks of reactive gliosis following neuronal injury, as well as microglial activation and metabolic stress; however, the mechanism of progression has yet to be fully elucidated [3,26,27]. In the brain, progressive neurological damage following ischemic insult is thought to be largely due to neuroinflammatory processes, in which dysregulated cytokine, neurotrophic factor, and protease production is thought to play a major part [28-30]. Gal-3, a cytokine with β-galactoside-binding properties, has been implicated in glial neuroinflammatory activity in the brain. In rats, it has been found to be significantly upregulated in microglia in the brain following focal ischemia, where it contributes to post-ischemic tissue remodeling [12,31]. Similarly, several retinal degenerative diseases associated with GFAP elevation and gliosis in Müller cells, including diabetic retinopathy and light-induced retinal degeneration, have also been linked to an increased expression of Gal-3 [14,15,32]. In this study, we have for the first time shown that the absence of Gal-3 significantly attenuates glial reactivity during hypoperfusion-induced retinal degeneration and significantly preserves photoreceptors and ganglion cells, as well as the retinal laminar morphology.

### Retinal effects of chronic cerebral hypoperfusion

One of the most commonly used models to investigate the effects of chronic ischemia is to permanently occlude the common carotid arteries in rats, which drastically reduces cerebral flow [6,33]. However, in this model, it is unclear to which extent the retina becomes hypoperfused [3,6,25,34]. For this study, we chose mice from a pure C57BL/6 background, a strain, which due to its poorly developed circle of Willis, is highly susceptible to cerebral ischemia through carotid artery occlusion [34-37]. When comparing various species and mouse strains for suitability as models for ischemia, Yang *et al*. found C57BL/6 mice to show a high consistency in cerebral blood flow reduction and the resultant ischemic lesions [34].

Investigations of the retinal cellular effects of permanent carotid artery occlusion report reactive gliosis and progressive neuronal cell death, in which the ganglion cells are among the first affected [3,6,23,37]. Interestingly, transient retinal ischemia, induced by elevated intraocular pressure, results in similar progressive degeneration beginning with inner retinal neurons, where one study found 71% fewer optic axons 5 months after insult [38,39]. Primary pathological events causing neuronal damage during ischemic injury to the CNS, in addition to the loss of circulation, include excitotoxic glutamate release from neurons and glia [24]. In the eye, increased levels of glutamate have been found in the vitreous during retinal ischemia [40]. Excess glutamate is normally removed by Müller cells through glutamine synthetase (GS), thereby preventing excitotoxicity [41]. However, in the post-ischemic retina, the homeostatic functions of Müller cells, such as glutamate uptake, ion buffering, and water balance are reduced, which contributes to neuronal damage [24,41]. The sustained glial activation after ischemia and loss of normal retinal metabolic activity is thought to be responsible for the secondary, progressive neuronal cell death [24,25], although the precise mechanism behind this phenomenon has not been fully elucidated.

In our study, the coils reduced the blood flow through the common carotid arteries to approximately 70% of normal values [2]. After 17 weeks, WT mice displayed an upregulation of Gal-3 expression, a significant loss of ganglion cells (*P* < 0.001) and photoreceptors (*P* < 0.001) and disruption of horizontal cells and bipolar cells (*P* < 0.001). Evidence of remodeling was also seen, where the overall morphology revealed disorganization of the retinal lamination, and PKCpan-labeled bipolar cells displayed sprouting into the ONL, a behavior associated with photoreceptor degeneration [42]. Müller cell GS expression was significantly downregulated, an event associated with gliosis, suggesting impaired glutamate uptake. Indeed, GFAP expression, considered the hallmark of gliosis, was significantly increased, as was Iba1, indicating activation of both Müller cells and microglia [41,43]. Since Iba1 also labels blood-born macrophages and we have previously shown that bilateral carotid artery occlusion in CD57bl/6 mice causes macrophage infiltration in the brain, we cannot exclude a macrophage contribution also in the ischemic retina [44].

In contrast to the WT hypoperfused mice, Gal-3 KO counterparts displayed no significant neuronal loss or glial activation and maintained glial expression of GS. We can therefore report that the reduction of cerebral blood flow by 30% does not on its own appear to cause any significant retinal injury in the absence of Gal-3. The mechanism behind the activation and the subsequent progression of Müller cell and microglial gliosis after ischemic insult may involve a combination of factors and crosstalk between these two cell types and recruited macrophages, where our results suggest Gal-3 is a possible mediator.

### Retinal neuroinflammation and Gal3

Gal-3 has been found to act as a pro-inflammatory mediator and has been implicated in numerous inflammatory diseases. Gal-3 upregulation (up to 96-fold) has been reported during several retinal pathologies and is thought to be required for microglial activation following ischemia in the brain [8,11,14,15,45,46]. The microglial response to injury is extremely rapid, where cells become activated within minutes, and is thought to precede macroglial responses [23,26,27,47]. It has been suggested that in the injured retina, Müller cells release Gal-3 via unconventional secretion, where it can then function as a microglial phagocytic ligand [14,48,49]. Our group has recently shown that Gal-3 secreted by brain microglia can bind the TLR4 receptor and in an autocrine fashion increase detrimental inflammation [50]. Extracellular Gal-3 can act as a bridging molecule by simultaneously binding to Mer receptor tyrosine kinase (MerTK) and phagocytic prey, thereby stimulating phagocytosis [48,51,52]. Intracellular effects of Gal-3 include enhancing microglial phagocytic activity through the activation of phosphatidylinositol-3-kinase (PI3K) via K-Ras [48,53]. Gal-3 can be found in different subcellular compartments in a range of cell types, and together with its numerous post-translational modifications has been found to have a wide range of functions [50]. In this study, Iba1-labeled cells appear to be the most likely source of Gal-3; however, previous studies have shown Müller cells and astrocytes to be the main point of supply during retinal disease [14,54]. Double labelings of Gal-3 with Müller cell and astrocytic markers GFAP (activated Müller cells and astrocytes) and CRALBP revealed no colocalization. In a clinical setting, Gal-3 has been found in cerebrospinal fluid (CSF) of infants with hypoxic-ischemic brain injury after birth asphyxiation, where it was found to contribute to detrimental neuroinflammation [55]. This study evaluated the possible systemic production and deposition in CSF of Gal-3, as well as possible leakage of Gal-3 into the CSF through blood-brain barrier (BBB) dysfunction. No difference was observed in CSF protein content between asphyxiated infants and controls suggesting that there were no major contribution resulting from BBB damage. To assess systemic contribution of Gal-3 to the CSF, infants with septic inflammation were screened, with no difference to controls, indicating a local production of Gal-3 in the injured CNS [55]. Over time, the expression of Gal-3 remained elevated within the clinically important group with moderate encephalopathy, in similarity to our findings after 17 weeks. The mechanism of action in which Gal-3 contributes to the maintenance and progression of neuroinflammatory processes in this setting may therefore involve the secretion of Gal-3 from activated cells, resulting in induction of pro-inflammatory cytokines such as interleukin (IL)-1β, tumor necrosis factor (TNF) α, and IL-6, enhancing glial reactivity through a positive feedback cycle and thereby contributing to neuronal cell death [14,41,48,56-61].

In our study, Gal-3 expression was mainly found in large structures resembling debris, as well as in inclusions in adjacent Iba1-labeled cells of microglial morphology. The appearance of cellular inclusions has previously been described in macrophages in the brain, where Gal-3 has been found to be a signal for phagocytosis of apoptotic cells [48]. Similarly, the phagocytic activity of retinal activated microglia and astrocytes has been correlated to increased Gal-3 expression and ganglion cell death after glaucomatous injury [54]. In this setting, Gal-3 possibly mediates dysregulated phagocytosis and glial activation, which we have shown can be attenuated through Gal-3 KO. Gal-3 is therefore a promising target for pharmaceutical intervention to minimize debilitating neuroinflammatory degeneration in the retina after ischemic injury.

## Conclusion

We have shown that a long-term moderate reduction of cerebral blood flow through the common carotid arteries results in degeneration of several retinal neuronal subtypes, as well glial reactivity, in the mouse. In the absence of Gal-3, these reactions are attenuated, resulting in a preservation of retinal neurons and the laminar architecture. This suggests that Gal-3 may be a link between neuroinflammation and retinal gliosis. Our results suggest that the neuroinflammatory processes propagated by glial cells results in a neurotoxic environment after ischemic injury, leading to progressive retinal damage. The findings indicate that Gal-3 is a potential target for the prevention of post-ischemic retinal pathology and make Gal-3 an interesting candidate for further study in retinal degenerative disease.

## Additional file

Additional file 1:
**Confocal linescan-generated three-dimensional movie of Galectin-3-positive inclusions in and Iba1-labeled microglial cell in a wild type hypoperfused retina.** Three-dimensional image of an activated microglial cell with Galectin-3 labeled inclusions in a wild type hypoperfused retina. Confocal linescan-generated three-dimensional image of an Iba1-labeled microglia (green) containing Galectin-3-positive inclusions (red). Cell bodies labeled with DAPI (blue).

## Abbreviations

bFGF: basic fibroblast growth factor
Gal-3: Galectin 3
GCL: ganglion cell layer
GFAP: glial fibrillary acidic protein
GS: glutamine synthetase
IL: interleukin
INL: inner nuclear layer
IPL: inner plexiform layer
KO: knock-out
MerTK: Mer receptor tyrosine kinase
ONL: outer nuclear layer
OPL: outer plexiform layer
PI3K: phosphatidylinositol-3-kinase
PKCpan: protein kinase C pan
TNF: tumor necrosis factor
WT: wildtype

## References

[1] Kalesnykas G, Tuulos T, Uusitalo H, Jolkkonen J (2008). Neurodegeneration and cellular stress in the retina and optic nerve in rat cerebral ischemia and hypoperfusion models. Neuroscience.

[2] Shibata M, Ohtani R, Ihara M, Tomimoto H (2004). White matter lesions and glial activation in a novel mouse model of chronic cerebral hypoperfusion. Stroke.

[3] Yamamoto H, Schimdt-Kastner R, Hamasaki DI, Yamamoto H, Parel JM (2006). Complex neurodegeneration in retina following moderate ischemia induced by bilateral common carotid artery occlusion in Wistar rats. Exp Eye Res.

[4] Davidson CM, Pappas BA, Stevens WD, Fortin T, Bennett SA (2000). Chronic cerebral hypoperfusion: loss of pupillary reflex, visual impairment and retinal neurodegeneration. Brain Res.

[5] Stevens WD, Fortin T, Pappas BA (2002). Retinal and optic nerve degeneration after chronic carotid ligation: time course and role of light exposure. Stroke.

[6] Chidlow G, Holman MC, Wood JO, Casson RJ (2010). Spatiotemporal characterization of optic nerve degeneration after chronic hypoperfusion in the rat. Invest Ophtalmol Vis Sci.

[7] Kaja S, Yang SH, Wei J, Fujitani K, Liu R, Brun-Zinkernagel AM (2003). Estrogen protects the inner retina from apoptosis and ischemia-induced loss of Vesl-1L/Homer 1c immunoreactive synaptic connections. Invest Opthalmol Vis Sci.

[8] Shin T (2013). The pleiotropic effects of galectin-3 in neuroinflammation: a review. Acta Histochem.

[9] Jin R, Yang G, Li G (2010). Inflammatory mechanisms in ischemic stroke: role of inflammatory cells. J Leukoc Biol.

[10] Patel AR, Ritzel R, McCullough LD, Liu F (2013). Microglia and ischemic stroke: a double-edged sword. Int J Physiol Pathophysiol Pharmacol.

[11] Lalancette-Hébert M, Swarup V, Beaulieu JM, Bohacek I, Abdelhamid E, Weng YC (2012). Galectin-3 is required for resident microglia activation and proliferation in response to ischemic injury. J Neurosci.

[12] Yan YP, Lang BT, Vemuganti R, Dempsey RJ (2009). Galectin-3 mediates post-ischemic tissue remodeling. Brain Res.

[13] Boza-Serrano A, Reyes JF, Rey NL, Leffler H, Bousset L, Nilsson U (2014). The role of Galectin-3 in α-synuclein-induced microglial activation. Acta Neuropathol Commun.

[14] Uehara F, Ohba N, Ozawa M (2001). Isolation and characterization of galectins in the mammalian retina. Invest Ophthalmol Vis Sci.

[15] Canning P, Glenn JV, Hsu DK, Liu FT, Gardiner TA, Stitt AW (2007). Inhibition of advanced glycation and absence of galectin-3 prevent blood-retinal barrier dysfunction during short-term diabetes. Exp Diabetes Res.

[16] Colnot C, Fowlis D, Ripoche MA, Bouchaert I, Poirier F (1998). Embryonic implantation in 1/galectin 3 double mutant mice. Dev Dyn.

[17] Engelsberg K, Ghosh F (2007). Transplantation of cultured adult porcine neuroretina. Cell Transpl.

[18] Taylor L, Arnér K, Engelsberg K, Ghosh F (2013). Effects of glial cell line-derived neurotrophic factor on the cultured adult full- thickness porcine retina. Curr Eye Res.

[19] Taylor L, Moran D, Arnér K, Warrant E, Ghosh F (2013). Stretch to see: lateral tension strongly determines cell survival in long-term cultures of adult porcine retina. IOVS.

[20] Taylor L, Arnér K, Taylor IH, Ghosh F (2014). Feet on the ground: physical support of the inner retina is a strong determinant for cell survival and structural preservation in vitro. IOVS.

[21] Taylor L, Arnér K, Ghosh F (2014). First responders: dynamics of pre-gliotic Müller. Cell responses in the isolated adult rat retina. Curr Eye Res.

[22] Lewis GP, Linberg KA, Fisher SK (1998). Neurite outgrowth from bipolar and horizontal cells after experimental retinal detachment. Invest Ophthalmol Vis Sci.

[23] Farkas E, Luiten PG, Bari F (2007). Permanent, bilateral common carotid artery occlusion in the rat: a model for chronic cerebral hypoperfusion-related neurodegenerative diseases. Brain Res Rev.

[24] Osborne NN, Casson RJ, Wood JP, Chidlow G, Graham M, Melena J (2004). Retinal ischemia: mechanisms of damage and potential therapeutic strategies. Prog Retin Eye Res.

[25] Osborne NN, Block F, Sontag KH (1991). Reduction of ocular blood flow results in glial fibrillary acidic protein (GFAP) expression in rat retinal Müller cells. Vis Neurosci.

[26] Sivilia S, Guiliani A, Fernández M, Turba ME, Forni M, Massella A (2009). Intravitreal NGF administration counteracts retina degeneration after permanent carotid artery occlusion in rat. BMC Neurosci.

[27] Barnett N, Osbourne N (1995). Prolonged bilateral carotid artery occlusion induces electrophysiological and immunohistochemical changes to the rat retina without causing histological damage. Exp Eye Res.

[28] Rosenberg GA (2002). Matrix metalloproteinases in neuroinflammation. Glia.

[29] Kim JB, Sig Choi J, Yu YM, Nam K, Piao CS, Kim SW (2006). HMGB1, a novel cytokine-like mediator linking acute neuronal death and delayed neuroinflammation in the postischemic brain. J Neurosci.

[30] Candelario-Jalil E, Yang Y, Rosenberg GA (2009). Diverse roles of matrix metalloproteinases and tissue inhibitors of metalloproteinases in neuroinflammation and cerebral ischemia. Neuroscience.

[31] Wesley UV, Vemuganti R, Ayvaci ER, Dempsey RJ (2013). Galectin-3 enhances angiogenic and migratory potential of microglial cells via modulation of integrin linked kinase signaling. Brain Res.

[32] Kim J, Moon C, Ahn M, Joo HG, Jin JK, Shin T (2009). Immunohistochemical localization of galectin-3 in the pig retina during postnatal development. Mol Vis.

[33] Spertus AD, Slakter JS, Weissman SS, Henkind P (1984). Experimental carotid occlusion: funduscopic and fluorescein angiographic findings. Br J Ophthalmol.

[34] Yang G, Kitagawa K, Matsushita K, Mabuchi T, Yagita Y, Yanagihara T (1997). C57BL/6 strain is most susceptible to cerebral ischemia following bilateral common carotid occlusion among seven mouse strains: selective neuronal death in the murine transient forebrain ischemia. Brain Res.

[35] Olsson T, Wieloch T, Smith ML (2003). Brain damage in a mouse model of global cerebral ischemia. Effect of NMDA receptor blockade. Brain Res.

[36] Olsson T, Hansson O, Nylandsted J, Jäättelä M, Smith ML, Wieloch T (2004). Lack of neuroprotection by heat shock protein 70 overexpression in a mouse model of global cerebral ischemia. Exp Brain Res.

[37] Kim BJ, Braun TA, Wordinger RJ, Clark AF (2013). Progressive morphological changes and impaired retinal function associated with temporal regulation of gene expression after retinal ischemia/reperfusion injury in mice. Mol Neurodegener.

[38] Danylkova NO, Pomeranz HD, Alcala SR, McLoon LK (2006). Histological and morphometric evaluation of transient retinal and optic nerve ischemia in rat. Brain Res.

[39] Hirrlinger PG, Ulbricht E, Iandiev I, Reichenbach A, Pannicke T (2010). Alterations in protein expression and membrane properties during Müller cell gliosis in a murine model of transient retinal ischemia. Neurosci Lett.

[40] Izumi Y, Hammerman Seth B, Kirby Charity O, Benz Ann M, Olney John W, Zorumski CF (2003). Involvement of glutamate in ischemic neurodegeneration in isolated retina. Vis Neurosci.

[41] Bringmann A, Pannicke T, Grosche J, Francke M, Wiedemann P, Skatchkov SN (2006). Müller cells in the healthy and diseased retina. Prog Retin Eye Res.

[42] Wang Q-P, Jammoul F, Duboc A (2008). Treatment of epilepsy: the GABA-transaminase inhibitor, vigabatrin, induces neuronal plasticity in the mouse retina. Eur J Neurosci.

[43] Ito D, Tanaka K, Suzuki S, Dembo T, Fukuuchi Y (2001). Enhanced expression of Iba1, ionized calcium-binding adapter molecule 1, after transient focal cerebral ischemia in rat brain. Stroke.

[44] Lambertsen KL, Deierborg T, Gregersen R, Clausen BH, Wirenfeldt M, Nielsen HH (2011). Differences in origin of reactive microglia in bone marrow chimeric mouse and rat after transient global ischemia. J Neuropathol Exp Neurol.

[45] Gerhardinger C, Costa MB, Coulombe MC, Toth I, Hoehn T, Grosu P (2005). Expression of acute-phase response proteins in retinal Müller cells in diabetes. Invest Ophthalmol Vis Sci.

[46] Zacks DN (2009). Gene transcription profile of the detached retina (an AOS thesis). Trans Am Ophthalmol Soc.

[47] Rotshenker S (2009). The role of Galectin-3/MAC-2 in the activation of the innate- immune function of phagocytosis in microglia in injury and disease. J Mol Neurosci.

[48] Caberoy NB, Alvarado G, Bigcas JL, Li W (2012). Galectin-3 is a new MerTK-specific eat-me signal. J Cell Physiol.

[49] Hughes RC (1999). Secretion of the galectin family of mammalian carbohydrate-binding proteins. Biochim Biophys Acta.

[50] Burguillos MA, Svensson M, Schulte T, Boza-Serrano A, Garcia-Quintanilla A, Kavanagh E (2015). Microglia-secreted galectin-3 acts as a toll-receptor 4 ligand and contributes to microglial activation. Cell Reports.

[51] Ravichandran KS, Lorenz U (2007). Engulfment of apoptotic cells: signals for a good meal. Nat Rev Immunol.

[52] Caberoy NB, Maiguel D, Kim Y, Li W (2010). Identification of tubby and tubby-like protein 1 as eat-me signals by phage display. Exp Cell Res.

[53] Rotshenker S, Reichert F, Gitik M, Haklai R, Elad-Sfadia G, Kloog Y (2008). Galectin-3/MAC-2, Ras and PI3K activate complement receptor-3 and scavenger receptor-AI/II mediated myelin phagocytosis in microglia. Glia.

[54] Nguyen JV, Soto I, Kim KY, Bushong EA, Oglesby E, Valiente-Soriano FJ (2011). Myelination transition zone astrocytes are constitutively phagocytic and have synuclein dependent reactivity in glaucoma. Proc Natl Acad Sci U S A.

[55] Sävman K, Heyes MP, Svedin P, Karlsson A (2013). Microglia/macrophage-derived inflammatory mediators galectin-3 and quinolinic acid are elevated in cerebrospinal fluid from newborn infants after birth asphyxia. Transl Stroke Res.

[56] Wang M, Wong WT (2014). Microglia-Müller cell interactions in the retina. Adv Exp Med Biol.

[57] Harada T, Harada C, Kohsaka S, Wada E, Yoshida K, Ohno S (2002). Microglia-Müller glia cell interactions control neurotrophic factor production during light-induced retinal degeneration. J Neurosci.

[58] Block ML, Hong JS (2005). Microglia and inflammation-mediated neurodegeneration: multiple triggers with a common mechanism. Prog Neurobiol.

[59] Wang M, Ma W, Zhao L, Fariss RN, Wong WT (2011). Adaptive Müller cell responses to microglial activation mediate neuroprotection and coordinate inflammation in the retina. J Neuroinflammation.

[60] Jeon SB, Yoon HJ, Chang CY, Koh HS, Jeon SH, Park EJ (2010). Galectin-3 exerts cytokine-like regulatory actions through the JAK-STAT pathway. J Immunol.

[61] Boje KM, Arora PK (1992). Microglial-produced nitric oxide and reactive nitrogen oxides mediate neuronal cell death. Brain Res.

